# The Field of Science

**Published:** 1894-03-10

**Authors:** 


### THE FIELD OF SCIENCE.

A remarkable electrical experiment has been demonstrated recently at the College of Physicians and Surgeons at Baltimore. A small electric light was introduced into the stomach of a patient by Professor Julius Friedenwald, who conducted the experiment. It has hitherto been considered impossible to bring this about, but in this instance over 200 people witnessed the experiment. The workings of the patient's internal organs became visible through the transparency created by the light.

The transmissibility of tuberculosis through fish has been attracting the attention of Professor Combermale of the Lille Faculty of Medicine. His investigations on this head pretty clearly show that it is not due to this particular food (on which they mainly subsist) that whole families in Iceland and in Canada succumb to phthisis; certainly it appears it is not through infection conveyed by this means. Professor Combermale has tried many methods to ascertain definite facts on this subject. For instance, he shows us that the bacilli in carp, which were inoculated with tubercle germs, can but rarely be demonstrated. Again, fish fed with substances containing tubercle germs, and living in water infected with tubercular sputa, have never become tuberculous, declares the investigator.

Tea and coffee are claiming a brief in their defence at the hands of Dr. Schultz-Schulzenstein, a German scientist, who has taken up the cause of these muchly-maligned beverages. This chemist has put into practical demonstration what all of us have long known, that neither tea nor coffee taken in reasonable proportions and drunk soon after they are made have any injurious effect upon us, granted, of course, we are in possession of good health. Dr. Schulzenstein carried out his investigations by preparing an extract resembling gastric juice, submitting to its action the albumen of a boiled egg. At the end of eight hours 94 per cent. of the albumen was digested. Then he added decoctions of tea and coffee to portions of the same mixture, and found at the end of the same time that in the case of coffee only 61 per cent., and in the case of tea only 66 per cent. of the albuminous contents was digested. This is sufficiently conclusive proof that coffee and tea, boiled so as to make a strong decoction, are both serious checks upon the process of digestion. At the same time this is not the case when the leaves or berries are submitted to the action of boiling water for but a few minutes. In both of these beverages there are to be discovered three elements—caffein or thein, aromatic substances, and tannin; it is this latter which has the deterrent effect upon digestion, an unwholesome element which is only dissolved after considerable steeping.

The question of the relationship between epilepsy and errors of refraction in the eye has been gone into by Mr. H. Work Dodd, whose results appeared in a recent contemporary. The eyes of one hundred cases of true epilepsy were examined for this purpose, and the refractions compared with those of apparently normal eyes. It appears that of simple hypermetropia there were 28 cases per cent. less in the epileptic than in the apparently normal class; of astigmatism of all kinds there were 26 cases per cent. more in the epileptic division than in the normal one. Mr. Dodd is led to conclude, through these and other differences, that given a certain condition of irritability of the nervous system—(1) The errors of refraction may excite epilepsy; (2) the correction of the errors of refraction will, in combination with other treatment, in many cases cure or relieve the epileptic condition; and (3) in some cases, when the refraction error has been corrected, the epilepsy will continue generally in a modified form, in consequence of other irritation, even though the error of refraction may have been the exciting cause of the fits in the first instance.

The report on Mr. Laws' investigation of the air in London sewers has been presented to the County Council, at whose instigation the investigations were made, in the hope of tracing further facts concerning the distribution of zymotic disease. The principal experiments were made in a sewer which runs under the Green Park, and the air of which has had sufficient time to become thoroughly impregnated, since it was constructed over 120 years ago. Mr. Laws first succeeded in estimating the percentage of carbonic acid gas, and then directed further attention to determining the microbial contents of sewer air. His results only go to confirm previous investigations on the same lines—that very few organisms are generally contained in sewer air; fewer, in fact, than are to be found in the freer outer air! An article which has just appeared in *Nature* gives further facts concerning this most interesting subject, and refers us to Dr. Petrie's observations on this same matter, which are well worth quoting. Dr. Petrie, on his part, examined no less than 100 samples of air in a Berlin sewer, and found on one occasion no organisms at all; and in another experiment only one bacterium was traced and three moulds. It appears, therefore, curious as it may seem, that drain air, as regards freedom from microbes, is very frequently superior to that which we inhale in our houses, and indeed compares especially favourably in this respect with the air in crowded reception-rooms. The conclusion of Mr. Laws' report says that although the organisms of sewer air do not probably constitute any source of danger, yet the latter may contain some poisonous chemical substance which is capable of producing a profound effect upon the general vitality.

